# Cytoskeletal Stability in the Auditory Organ *In Vivo*: RhoA Is Dispensable for Wound Healing but Essential for Hair Cell Development

**DOI:** 10.1523/ENEURO.0149-17.2017

**Published:** 2017-09-18

**Authors:** Tommi Anttonen, Ilya Belevich, Maarja Laos, Anni Herranen, Eija Jokitalo, Cord Brakebusch, Ulla Pirvola

**Affiliations:** 1 Department of Biosciences, Division of Physiology and Neuroscience, University of Helsinki, Helsinki, Finland; 2 Institute of Biotechnology, University of Helsinki, Helsinki, Finland; 3 Biomedical Institute, Biotech Research and Innovation Centre, University of Copenhagen, Copenhagen, Denmark

**Keywords:** Auditory, cytoskeleton, development, inner ear, RhoA, wound healing

## Abstract

Wound healing in the inner ear sensory epithelia is performed by the apical domains of supporting cells (SCs). Junctional F-actin belts of SCs are thin during development but become exceptionally thick during maturation. The functional significance of the thick belts is not fully understood. We have studied the role of F-actin belts during wound healing in the developing and adult cochlea of mice *in vivo*. We show that the thick belts serve as intracellular scaffolds that preserve the positions of surviving cells in the cochlear sensory epithelium. Junctions associated with the thick F-actin belts did not readily disassemble during wound healing. To compensate for this, basolateral membranes of SCs participated in the closure of surface breach. Because not only neighboring but also distant SCs contributed to wound healing by basolateral protrusions, this event appears to be triggered by contact-independent diffusible signals. In the search for regulators of wound healing, we inactivated *RhoA* in SCs, which, however, did not limit wound healing. *RhoA* inactivation in developing outer hair cells (OHCs) caused myosin II delocalization from the perijunctional domain and apical cell-surface enlargement. These abnormalities led to the extrusion of OHCs from the epithelium. These results demonstrate the importance of stability of the apical domain, both in wound repair by SCs and in development of OHCs, and that only this latter function is regulated by *RhoA*. Because the correct cytoarchitecture of the cochlear sensory epithelium is required for normal hearing, the stability of cell apices should be maintained in regenerative and protective interventions.

## Significance Statement

We show that the stability of the apical domain is required for the development of cochlear hair cells and that it is regulated by the GTPase RhoA. We also show that the apical stability of supporting cells is critical for wound healing in the adult cochlea, as it maintains cellular topology and dimensions at the surface of the sensory epithelium. Surprisingly, although RhoA is known to regulate wound healing in several cell contexts, the wound repair process in the cochlear sensory epithelium does not depend on it. Our results point to the importance of preservation of apical stability of cochlear sensory epithelial cells in future therapeutic interventions aiming at treating hearing loss.

## Introduction

The mammalian auditory sensory epithelium of the cochlea, the organ of Corti, comprises sensory hair cells (HCs) and nonsensory supporting cells (SCs). At its apical surface, four rows of HCs with their stereociliary bundles and intervening SCs form an intricate checkerboard-like pattern (Jahan et al., 2015). HCs transduce and amplify sound information, whereas SCs provide structural support and perform homeostatic and reparative functions. Of the two types of auditory HCs, outer hair cells (OHCs) are more vulnerable to trauma than inner hair cells. After traumas causing OHC loss, such as acoustic overexposure and ototoxic drugs, SCs reseal the epithelial surface breach and thereby are critical to prevent progressive HC loss. This process is referred to as wound healing or scar formation in the organ of Corti (Raphael and Altschuler, 1991). Of the several types of auditory SCs, Deiters cells and pillar cells are the critical ones in wound healing. Upon OHC loss, apices of these SCs expand and close the lesion with new SC–SC junctions. (Forge, 1985; Raphael and Altschuler, 1991; Leonova and Raphael, 1997; Anttonen et al., 2012, 2014; Taylor et al., 2012). Actin accumulates to the new junction and integrates into the junctional F-actin belt. SCs normally prevent the collapse of the organ of Corti after HC loss. However, after extended posttrauma periods, the organ of Corti is often replaced by a flat epithelium. The flat epithelium appears to be composed at least in part of cells located laterally to the organ of Corti that replace degenerated pillar and Deiters cells (Raphael et al., 2007; Taylor et al., 2012; Oesterle, 2013; Kersigo and Fritzsch, 2015).

The F-actin belts of SCs of the inner ear have drawn special attention. Their function has been mostly studied in the sensory epithelium of the utricle, one of the vestibular organs. Although cytoarchitectural features are simpler in the vestibular sensory epithelia, their SCs share many basic functions with those of the organ of Corti. In mammals, mature SCs possess thick F-actin belts, as opposed to the thin belts of developing SCs. The thin belts together with lower amounts of the adherens junction protein E-cadherin have been suggested to confer proliferative capacity of developing SCs. Consistently, the steep decline in SCs’ proliferative capacity during the juvenile life correlates with the thickening of their F-actin belts (Meyers and Corwin, 2007; Burns et al., 2008; Collado et al., 2011; Burns and Corwin, 2014). Live-imaging studies with utricular explants during wound healing have shown that the overall actin turnover rate is considerably lower in thick-belted SCs compared with thin-belted SCs (Burns and Corwin, 2014). This suggests that thin belts are more plastic during repair than thick belts. The significance of the thick F-actin belts of mature auditory SCs is poorly understood. Therefore, to study this further, we have induced lesions in the developing and adult organ of Corti *in vivo* by using genetic and pharmacological methods. The adult organ of Corti is not amenable for *in vitro* culturing. Also, *in vivo* approaches have the advantage that morphologic changes generated by wound healing are not masked by those produced by *in vitro* culture conditions.

Regarding the regulation of the apical actomyosin network of the cells of the organ of Corti, previous gene inactivation studies have demonstrated that the prototypical members of the Rho family of small guanosine 5′-triphosphatases (small GTPases) Rac1 (Grimsley-Myers et al., 2009) and Cdc42 (Anttonen et al., 2012, 2014; Ueyama et al., 2014; Kirjavainen et al., 2015) regulate cytoskeletal development. At least in the case of Cdc42, the actin cytoskeleton was primarily affected. The third member of the Rho family is the ubiquitously expressed RhoA. Major effectors of the RhoA pathway are the perijunctional actomyosin network and associated cell-cell contacts. In general, RhoA/Rho-associated kinase (ROCK) signaling regulates assembly of nonmuscle myosin II (NMII) on actin filaments and stimulates actomyosin contractility. Signaling by RhoA and the formin mDia promotes F-actin polymerization. RhoA signaling regulates diverse cellular events, such as wound repair, migration, cytokinesis, and morphogenesis (Clark et al., 2009; Pedersen and Brakebusch, 2012; Martin and Goldstein, 2014). The role of RhoA in the cells of the organ of Corti has not yet been studied with genetic approaches. To understand whether and how it regulates cytoskeletal development and wound healing in this sensory epithelium, we have analyzed the effects of *RhoA* inactivation in both auditory SCs and OHCs.

## Materials and Methods

### Mice

Mice homozygous for the floxed *RhoA* allele (*RhoA^fl/fl^*, Jackson et al., 2011) were crossed with mice carrying the *Fgfr3-iCre-ER^T2^* transgene (Young et al., 2010) to obtain *RhoA^fl/fl^;Fgfr3-iCre-ER^T2^* animals. These mice and control *RhoA^fl/fl^* mice from the same litters were analyzed at embryonic day 18.5 (E18.5), postnatal day 20 (P20), and P50 (recombination paradigms described below). Genotyping by PCR was conducted as previously described (Young et al., 2010; Jackson et al., 2011). *Gfi1^GFP/GFP^* knock-in mice (growth factor independent 1) and control littermates were analyzed at E18.5. Generation and genotyping of these mutant animals have been described (Yücel et al., 2004). Timed pregnancies were established by the detection of a vaginal plug, with noon on the day of a plug defined as E0.5. Both females and males were used in the analysis. Mouse lines were maintained in a mixed background. The ICR strain was used for studies of adult mice. All animal work was conducted according to relevant national and international guidelines. Approval for animal experiments was obtained from the National Animal Experiment Board.

### Ototoxic trauma

OHC loss was induced at P20 by a single subcutaneous injection of 1 mg/g kanamycin (Sigma-Aldrich) followed by a single intraperitoneal injection of 0.4 mg/g furosemide (Fresenius Kabi), according to an established protocol (Oesterle et al., 2008; Taylor et al., 2008; Anttonen et al., 2012; 2014). This trauma model is termed KAFU treatment in the figures. The interval between the injections was 30 min. Animals were killed 36 h or 9 d postlesion. In the case of *RhoA^fl/fl^;Fgfr3-iCre-ER^T2^* mutant mice treated with tamoxifen (Sigma-Aldrich) at P2 and P3, the same regimen of ototoxic trauma was applied at P20.

### Conditional and inducible *RhoA* inactivation

To induce embryonic inactivation of *RhoA* in OHCs and SCs, pregnant *RhoA^fl/fl^;Fgfr3-iCre-ER^T2^* mice were injected intraperitoneally with 3 mg tamoxifen at E13 and E14. The characteristics of *iCre*-mediated recombination in the cochlea with this injection paradigm have been described (Kirjavainen et al., 2015). Litters were killed and embryos dissected at E18.5. For postnatal *RhoA* inactivation in auditory SCs, *RhoA^fl/fl^;Fgfr3-iCre-ER^T2^* mice were injected intraperitoneally with 50 μg/g tamoxifen at P2 and P3 or P16 and P17, as previously described (Anttonen et al., 2012). Recombination characteristics are also described in that prior publication. Animals were killed and cochleas fixed at P18 or P50.

### Immunohistochemistry on paraffin sections

Cochleas were perilymphatically fixed with 4% paraformaldehyde (PFA) in PBS and immersed in the fixative overnight at 4°C. Cochleas from adult mice were decalcified in 0.5 m EDTA, pH 7.5. Cochleas were embedded into paraffin (Paraplast, Thermo Fisher Scientific). 5-μm-thick sections were cut in midmodiolar plane through cochleas. After deparaffinization, epitopes were unmasked by microwave heating (900 W) in 10 mm citrate buffer, pH 6.0, for 10 min of boiling. Sections were blocked for 30 min with 10% goat serum (Jackson ImmunoResearch) in PBS containing 0.25% Triton X-100 (PBS-T). Incubation with primary antibodies diluted in PBS-T was performed for 48 h at 4°C. The following primary antibodies were used: rabbit polyclonal β-tubulin (Abcam), goat polyclonal prestin (Santa Cruz Biotechnology), rabbit monoclonal cleaved caspase-3 (Cell Signaling Technology), rabbit polyclonal espin (Zheng et al., 2000), rabbit monoclonal Ki-67 (LabVision/Thermo Fisher Scientific), rabbit polyclonal myosin 6 (Proteus Biosciences), and mouse monoclonal nonerythroid spectrin (fodrin; Ylikoski et al., 1992). Detection was performed with the Vectastain Elite ABC kit or Vectastain Mouse-On-Mouse kit and the diaminobenzidine substrate kit (all from Vector Laboratories). Sections were counterstained with 3% methyl green and mounted in Permount (Thermo Fisher Scientific). A part of consecutive sections was stained with hematoxylin (Shandon Instant Hematoxylin, Thermo Fisher Scientific). For all antibodies used in paraffin sections, a minimum of four cochleas per age/postlesion time point/genotype were prepared.

### Whole-mount specimens

Adult cochleas fixed with PFA and decalcified with EDTA were cut in midmodiolar plane in half, such that the coiled organ of Corti was separated into four pieces. These pieces were cleaned from the surrounding tissue, and the tectorial membrane was removed. E18.5 cochleas were fixed with PFA, and the organ of Corti was dissected as one whole specimen. For immunofluorescence, whole-mount specimens were blocked for 30 min with 10% donkey serum (Jackson ImmunoResearch) in PBS-T, followed by incubation with primary antibodies in PBS-T for 48 h at 4°C. The following primary antibodies were used: rat monoclonal E-cadherin, mouse monoclonal acetylated tubulin (both from Sigma-Aldrich), mouse monoclonal ZO-1 (Invitrogen/Thermo Fisher Scientific), rabbit polyclonal Vangl2 (Montcouquiol et al., 2006), and rabbit polyclonal NMIIb (Covance). Secondary antibodies conjugated to Alexa Fluor 568, 594, or 647 were used for detection. After antibody incubations, F-actin filaments were visualized using Oregon Green 514–conjugated phalloidin (1:400 in PBS-T). Nuclei were stained with DAPI. ProLong Gold antifade reagent was used for mounting (all from Invitrogen/Thermo Fisher Scientific). A minimum of four cochlear whole-mount specimens per postlesion time point and genotype were prepared for phalloidin labeling and antibody stainings.

### Light microscopy, data analysis, and image processing

Sections were analyzed with bright-field optics under a BX61 microscope equipped with UPlanApo 10×, 20×, and 60× objectives. Images were acquired through a DP73 CCD color camera and CellSens software (all from Olympus). Confocal images were acquired using a Leica TCS SP5 laser scanning microscope with 63×/1.3-NA glycerol objective. The acquisition software was Leica LAS AF. Image analysis and *z*-projections of light microscopy data were done with ImageJ (National Institutes of Health) equipped with Bio-Formats Importer plugin (Open Microscopy Environment).

### Specimen preparation for serial block-face scanning electron microscopy

Contralateral cochleas were processed for serial block-face scanning electron microscopy (SBEM). In short, cochleas were fixed by perilymphatic perfusion with 2.5% glutaraldehyde in 0.1 m phosphate buffer, pH 7.4. Specimens were immersed in this fixative overnight at 4°C. The organ of Corti was microdissected from the surrounding tissue, and areas of interest were selected under differential interference contrast (DIC) optics. In the case of ototoxically lesioned adult cochleas, the medial turn was used for analysis. Selected pieces were stained with heavy metals, dehydrated, and embedded in Durcupan ACM resin (Fluka/Sigma-Aldrich).

### SBEM data acquisition, image processing, 3D reconstruction, and analysis

The processing, data acquisition, image processing, 3D reconstruction, and analysis of SBEM data are described in detail in Anttonen et al. (2014). In short, resin-embedded specimens were trimmed, mounted on a 3View pin using conductive cyanoacrylate glue (CircuitWorks, Chemtronics), and covered with silver paint (Agar Scientific). The tissue block was coated with a 5-nm-thick layer of platinum in a sputter coater (Quorum Q150TS; Quorum Technologies). Specimens were imaged on an FEI Quanta 250 FEG scanning electron microscope equipped with a 3View system (Gatan). The cutting *z*-step was 40 nm. Specimens were imaged with the final pixel size of 20 nm. Obtained images were further processed in Microscopy Image Browser (Belevich et al., 2016). 3D representations of data were segmented using Amira (Visage Imaging) and Microscopy Image Browser and further rendered in Amira.

### Measurements of the reticular lamina length and cell surface area

**Figure 1.**
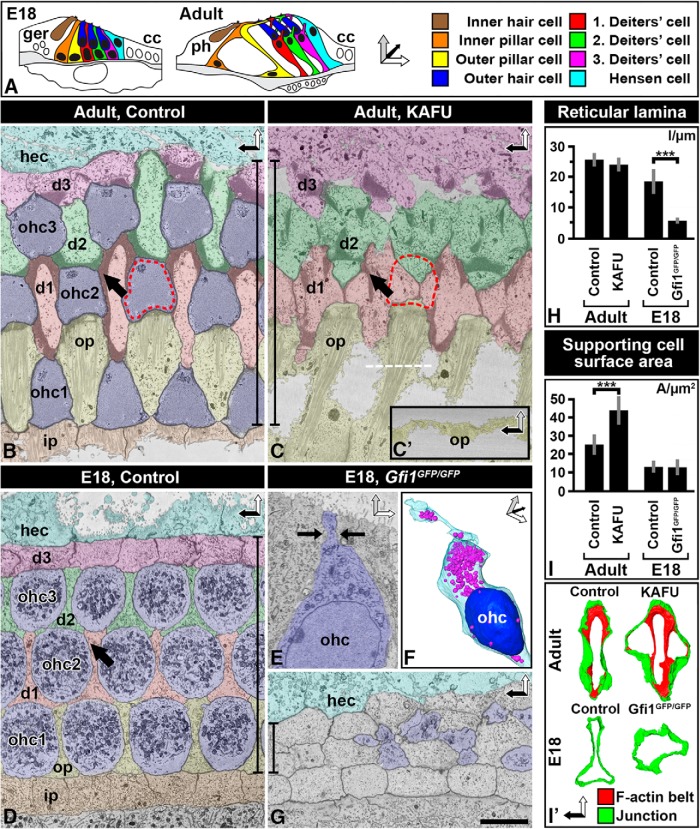
Thick F-actin belts of mature supporting cells maintain spatial organization at the surface of the lesioned organ of Corti. ***A***, Illustrations of the developing and adult organ of Corti. Coordinate arrows demonstrate image orientations. Color coding is shown on the right. ***B***, ***C***, SBEM single block-face images at the level of the apical cell junctions of the adult (P20) organ of Corti of the medial cochlear turn. In the nonlesioned cochlea, OHCs (blue) are evenly delineated by SCs. Arrow marks a thick F-actin belt of Deiters cells (***B***). 1.5 d after induction of ototoxic trauma (KAFU, see Methods), thick-belted SCs have resealed the epithelial surface (red hatched line in ***C***). The line demonstrates that the length of the reticular lamina is maintained, similar to positioning of survived cells. White dashed line shows orientation in ***C′***. ***C′***, Side view of the scar between outer pillar cells. ***D–G***, In the developing (E18.5) organ of Corti, Deiters cells have thin belts (arrow; ***D***). A degenerating OHC from a *Gfi1^GFP/GFP^* mouse cochlea is undergoing apical constriction (arrows), a typical feature of degenerating hair cells (***E***). A degenerating OHC from a *Gfi1^GFP/GFP^* mouse shown in 3D, with colored mitochondria (purple) and nucleus (blue; ***F***). Upon OHC loss in *Gfi1^GFP/GFP^* mice, thin-belted Deiters cells collapse and the reticular lamina is shrunken (***G***). Compare the line in ***G*** demonstrating the length of the reticular lamina to the corresponding line in the image from the adult organ of Corti (***C***). ***H***, Quantification (mean ± SD) shows that OHC loss leads to significant reduction of the width of the reticular lamina during development, but not at adulthood. ***I***, ***I′***, Quantification (mean ± SD) shows that apices of developing Deiters cells do not expand, as opposed to mature Deiters cells (***I***). This is evidenced by modeling of F-actin belts (red) and junctions (green; ***I′***). Abbreviations: cc, Claudius cell; d, Deiters cell; ger, greater epithelial ridge; hec, Hensen cell; i.p., inner pillar cell; KAFU, kanamycin and furosemide; ohc, outer hair cell; op, outer pillar cell; SC, supporting cell; ph, phalangeal cell. Scale bar (in ***G***): ***B–E***, ***G***, 5 µm; ***F***, 7.5 µm; ***I′***, 6.5 µm.

In P20 wild-type mice exposed to ototoxins and analyzed 36 h postlesion and in E18.5 *Gfi1^GFP/GFP^* mice, the medial-to-lateral length of the reticular lamina was measured as the linear distance between the inner pillar cell-first OHC row junction and the Hensen cell-third Deiters cell row junction (Fig. 1*H*). Measurements were done from SBEM datasets using Amira and from confocal image stacks using ImageJ. The medial turn of cochlea was used for analysis.

In wild-type P20 cochleas challenged with ototoxins and analyzed 36 h postlesion and in E18.5 *Gfi1^GFP/GFP^* cochleas, the surface areas of Deiters cells, defined as the area delineated by the Deiters cell apical junctional domain, were measured from SBEM datasets using Amira and from confocal image stack using ImageJ (Fig. 1*I*). Measurements were done in the medial turn. SCs lacking all adjacent OHCs were used for analysis.

In E18.5 cochleas from *RhoA^fl/fl^;Fgfr3-iCre-ER^T2^* mice and from littermate control mice, the apical surface areas of OHCs, defined as the area delineated by the perijunctional domain, were measured from confocal image stacks with ImageJ (Fig. 6*H*). Measurements were done in the basal turn of cochlea before the OHC nuclei had risen to the epithelial surface.

*P* values were obtained with a two-tailed *t* test. *P* values <0.05 were considered significant. Numbers of specimens, mean values, standard deviations, and *P* values are listed in Table 1.

**Table 1.** Parameters used for quantification and statistical analysis.

Type and analysis	Cochleas	Cochleas total	Analysis area (µm)	Measurements/sample	Measurements total	*n*	Average	SD	Two-tailed *t* test
Reticular lamina length (µm)									
E18									1.17 × 10^–11^
Control		4				18	18.44	3.94	
SBEM	1		70	9	9				
Confocal	3		3 × 100	3	9				
Gfi1^GFP/GFP^		3				12	5.47	0.84	
SBEM	1		61	3	3				
Confocal	2		2 × 100	3	9				0.051
Adult									
Control		4				13	25.58	2.02	
SBEM	2		46; 75	3; 4	7				
Confocal	2		2 × 100	3	6				
KAFU		4				12	24.04	2.04	
SBEM	2		67; 75	3	6				
Confocal	2		2 × 100	3	6				
Deiters cell surface area (µm^2^)									
E18									0.41
Control		3				46	13.08	3	
SBEM	1		70	25	25				
Confocal	2		2 × 100	9;12	21				
Gfi1^GFP/GFP^		3				55	12.77	4.15	
SBEM	1		61	27	27				
Confocal	2		2 × 100	9;19	28				
Adult									1.23 × 10^–27^
Control		3				46	25.23	5.32	
SBEM	2		46; 75	10;16	26				
Confocal	1		100	20	20				
KAFU		4				60	43.97	7.55	
SBEM	2		67; 75	6;14	20				
Confocal	2		2 × 100	18;22	40				
Hair cell surface area (µm^2^)									
OHCs									1.90E-22
E18 control									
Confocal	6	50–120	19; 15; 17; 19; 31; 57	158	28.91	3.21			
E18, TM E13–14 RhoA^fl/fl^;Fgfr3-iCreER^T2^									
Confocal	7	50–120	10; 17; 14; 21; 7; 25; 33	127	45.62	15.72			
IHCs									0.61
E18 control									
Confocal	6	50–120	6; 5; 5; 6; 8; 19	49	36.1	4.81			
E18, TM E13–14 RhoA^fl/fl^;Fgfr3-iCreER^T2^									
Confocal	5	50–120	5; 6; 10; 10; 11	42	35.5	5.94			

## Results

### *In vivo* models to study the role of the thin and thick F-actin belts of supporting cells during wound healing

The basic cellular organization of the organ of Corti of the mouse is established at birth. However, F-actin belts of SCs are still thin at this age (Fig. 1*A–D*), allowing studies on wound healing mechanisms in the absence of thick belts. Because studies with organotypic explants are complicated by cell movement and flattening due to culturing, we have applied *in vivo* approaches. The Gfi1 transcription factor is required for OHC survival, as evidenced by extensive death of these cells around birth in *Gfi1* loss-of-function mutant mice (Wallis et al., 2003). Thus, wound healing by the thin-belted SCs can be studied *in vivo* in these mice at E18 (Yücel et al., 2004). For comparison, to study wound healing by the thick F-actin belts of adult SCs, we killed OHCs from the structurally mature organ of Corti at P20. To achieve this, we exposed mice to furosemide, a loop diuretic drug, and to kanamycin, an aminoglycoside antibiotic. The synergistic effect of these drugs causes a rapid OHC loss that progresses in a basal-to-apical turn gradient. Already 1 d postlesion, we found extensive OHC loss in the medial and basal turns of the cochlea, in accordance with previous studies (Oesterle et al., 2008; Taylor et al., 2008; Anttonen et al., 2012). Thus, although OHC loss is caused by different reasons in the *Gfi1* loss-of-function cochleas and the ototoxically challenged cochleas, in both cases these cells are rapidly eliminated in a basal-to-apical coil gradient that allows for analysis of the acute wound healing response by SCs.

### Thick F-actin belts are essential for the maintenance of dimensions and patterning at the surface of the organ of Corti

We first prepared SBEM datasets from the adult organ of Corti fixed acutely (1.5 d) after the induction of ototoxic lesion. Analysis of single block-face images showed that the width (medial-lateral) of the reticular lamina of the lesioned organ of Corti was comparable to that of the nonlesioned organ, despite OHC loss (Fig. 1*B*, *C*, *C′*, *H*). Apices of Deiters cells and outer pillar cells—the SC subtypes surrounding OHCs—maintained their positions although their apices had expanded to close the sites previously occupied by OHCs (Fig. 1*C*). These observations suggest that the thick F-actin belts of mature SCs may be required for the maintenance of the positioning of cell apices during wound healing.

To be able to compare wound healing at the two ages, we first confirmed that developing OHCs are eliminated in a similar fashion as described for adult OHCs (Forge, 1985; Raphael and Altschuler, 1991; Leonova and Raphael, 1997; Anttonen et al., 2014). Indeed, similar to degenerating adult OHCs, apices of E18 *Gfi1* mutant OHCs became constricted and their cell bodies degenerated within the organ of Corti (Fig. 1*E*, *F*). The reticular lamina remained closed during the elimination of OHCs, indicating that developing SCs readily reseal the epithelial surface. However, unlike at adulthood, OHC loss led to a dramatic decrease of the width of the reticular lamina (Fig. 1*D*, *G*, *H*). Normal shapes and positioning of SC apices were lost with a concomitant mispatterning of OHC rows (Fig. 1*G*). Further, apical domains of developing SCs did not expand, as opposed to apices of mature SCs (Fig. 1*I*, *I′*). Altogether, thick-belted SCs are able to maintain cellular topology at the surface of the lesioned organ of Corti while thin-belted SCs fail to do this.

### Junctional components do not readily detach from the thick F-actin belts

Although the apices of adult SCs need to be rigid to allow structural maintenance of the reticular lamina, they should be plastic enough to be remodeled upon wound healing. Thick F-actin belts of adult SCs are known to be exceptionally stable and motionless (Forge, 1985; Raphael and Altschuler, 1991; Leonova and Raphael, 1997; Burns et al., 2008; Anttonen et al., 2014; Burns and Corwin, 2014). This raises the question how they contribute to wound healing, which requires a certain level of structural plasticity. In the adult utricular SCs, actin remodeling is most dynamic at the side of the thick F-actin belt facing the membranous junctional domain (Burns and Corwin, 2014). Therefore, junctional components may readily detach from this dynamic F-actin belt domain and incorporate into a new SC–SC junctional contact.

**Figure 2.**
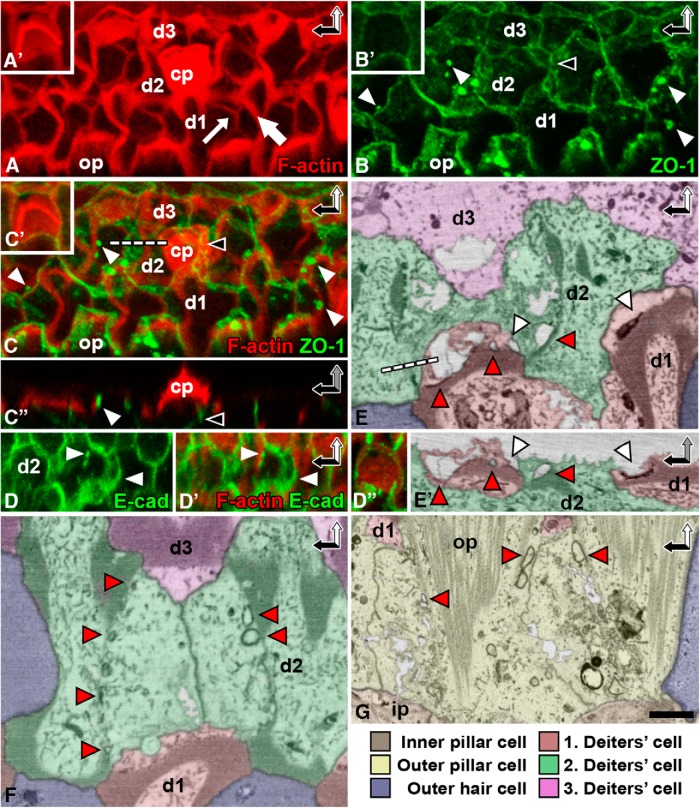
Junctional proteins of supporting cells are not uniformly recruited to new junctions after outer hair cell loss. The organ of Corti of P20 cochlea analyzed 1.5 d after induction of ototoxic trauma (see Methods). ***A–C′***, Confocal microscopy images show phalloidin-labeled original F-actin belts (thick arrow) as well as scar-forming thinner belts (thin arrow) at the site of lost OHCs. Double-labeling shows that ZO-1–positive junctional plaques (white arrowheads) remain associated with the original belts, despite formation of new junctions at the lesion site. Black arrowhead marks a new junction below the cuticular plate of a dead OHC. White dashed line shows orientation in ***C″***. Insets (***A′***, ***B′***, ***C′***) show respective double-labeling in a nonlesioned specimen. ***D***, ***D′***, Double-labeling for E-cadherin and phalloidin shows that the junctional plaques close to the original F-actin belt are also positive for E-cadherin (white arrowheads). ***D″***, Double-labeling for E-cadherin and phalloidin in a nonlesioned specimen. ***E***, ***E′***, Block-face single images confirm that Deiters cell junctions do not readily detach from the original belts during wound healing. Junctional plaques form vacuoles (red arrowheads) and surface depressions (white arrowheads). White dashed line shows orientation in ***E′***. ***F***, Block-face single image shows a typical cross-shaped scar formed by two Deiters cells. It also shows original F-actin belts with detached junctional plaques (red arrows). ***G***, Junctional components fail to readily detach from the original F-actin belts in scar-forming outer pillar cells as well (red arrowheads). Note also the prominent microtubule bundles in these cells. Color coding refers to block-face images. Abbreviations: d, Deiters cell; cp, cuticular plate; hec, Hensen cell, i.p., inner pillar cell; ohc, outer hair cell; op, outer pillar cell. Scale bars (in ***F***): ***A–C′***, 5 µm; ***D–D″***, 6.6 µm; ***E–G***, 1.6 µm.

As opposed to this expectation, our analysis made acutely (1.5 d) after ototoxic insult in the adult organ of Corti showed that Deiters cells contained multiple plaques of junctional proteins associated with the original belts. For this analysis, whole-mount specimens were immunolabeled for ZO-1, a tight junction–associated protein, and for E-cadherin. The specimens were colabeled with phalloidin to detect F-actin and viewed under confocal microscopy (Fig. 2*A–D″*). SBEM analysis confirmed that large electron-dense junctional plaques remained in contact with the belts (Fig. 2*E–G*). Some of the plaques formed grooves on the cell surface, and others formed internalized electron-dense sheets or vesicles (Fig. 2*E*, *E′*). Similar to Deiters cells, outer pillar cells displayed junctional domains that resisted detachment from the original F-actin belts (Fig. 2*G*). These results indicate that the original SC junctions and the associated membrane domains do not readily contribute to wound healing. This raises the question of where the membrane needed for lesion closure comes from.

### Supporting cells that lack junctional contacts with dying hair cells contribute to wound healing

**Figure 3.**
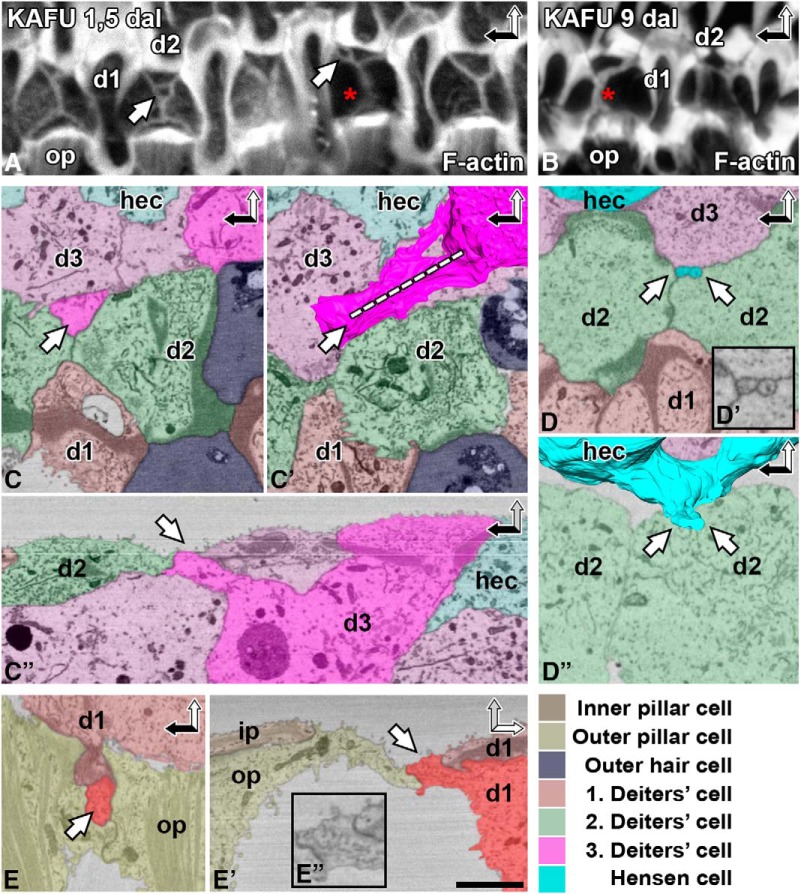
Supporting cells without junctional contacts to dying outer hair cells contribute to acute wound healing. The organ of Corti of P20 cochlea analyzed 1.5 and 9 d after ototoxic trauma (KAFU, see Methods). ***A***, ***B***, Phalloidin labeling at the acute postlesion stage shows that the scars formed by Deiters cells are associated with F-actin rings (white arrows; ***A***). Similar structures are not found at the later postlesion stage (***B***). Note the reinforcement of F-actin scars (red asterisks), evident by comparing the intensity of phalloidin labeling at the two postlesion time points. ***C–C″***, A ring-like structure (white arrow) is present at the scar. It originates from a basolateral extension of a distant Deiters cell, shown in a block-face single image (***C***) and after 3D modeling (***C′***). Dashed line shows orientation in ***C″***. ***D–D″***, A Hensen cell has extended two basolateral membrane protrusions to the scar (***D***), shown at a higher magnification in ***D′*** and with 3D modeling in ***D″***. ***E–E″***, A basolateral projection of a Deiters cell is found between two outer pillar cells, shown in two orientations (***E*** and ***E′***, ***E″***). Color coding refers to SBEM images. SC basolateral extensions are shown by more intense colors. Abbreviations: d, Deiters cell; dal, days after lesion; hec, Hensen cell; i.p., inner pillar cell; KAFU, kanamycin and furosemide; ohc, outer hair cell; op, outer pillar cell. Scale bar (in ***E′***): ***A***, ***B***, 5 µm; ***C***, ***D***, ***D″–E′***, 2.5 µm; ***D′***, ***E″***, 1.5 µm.

Although new membrane might be added from intracellular stores during wound healing, it could also be recruited from the basolateral membrane (Sheetz, 2001). Therefore, as a next step, we searched for evidence for the recruitment of SC basolateral membranes to lesion sites. The reticular lamina is typically sealed by SCs that share junctional contacts with lost OHCs. Deiters cells form a cross-shaped F-actin/junctional entity, referred to as scar (Fig. 1*C*). Interestingly, acutely (1.5 d) after the induction of ototoxicity, several scars showed deviations from the general cross-shaped morphology. Phalloidin labeling revealed that these scars were associated with ring-like actin structures of unknown origin (Fig. 3*A*). These odd structures were no longer found 9 d postlesion (Fig. 3*B*).

To elucidate the origin of the ring-like structures, we prepared SBEM datasets from the organs of Corti fixed acutely (1.5 d) after ototoxic lesion. Block-face single images revealed several ring-like structures that were continuous with the junctional scars (*n* = 5; Fig. 3*C*, *D*, *E*). 3D modeling demonstrated that the rings were formed by basolateral protrusions of Deiters cells and more distant Hensen cells. These protrusions extended within the organ of Corti to lesion sites at the surface (Fig. 3*C–E″*). These findings suggest that a preexisting connection between a SC and a dying OHC is not necessarily required for wound healing. Rather, SC basolateral membranes can be independently recruited to the lesion site. Taken together, our data provide direct evidence for two unique wound healing features by adult auditory SCs: uneven junctional disassembly from the old F-actin belt and the contribution of basolateral membrane to wound repair. These events have been previously suggested to occur based on immunofluorescence analysis (Raphael and Altschuler, 1991; Leonova and Raphael, 1997). However, SBEM coupled with 3D modeling provided direct evidence for these mechanisms.

### RhoA is dispensable for supporting cell maturation and for wound healing

**Figure 4.**
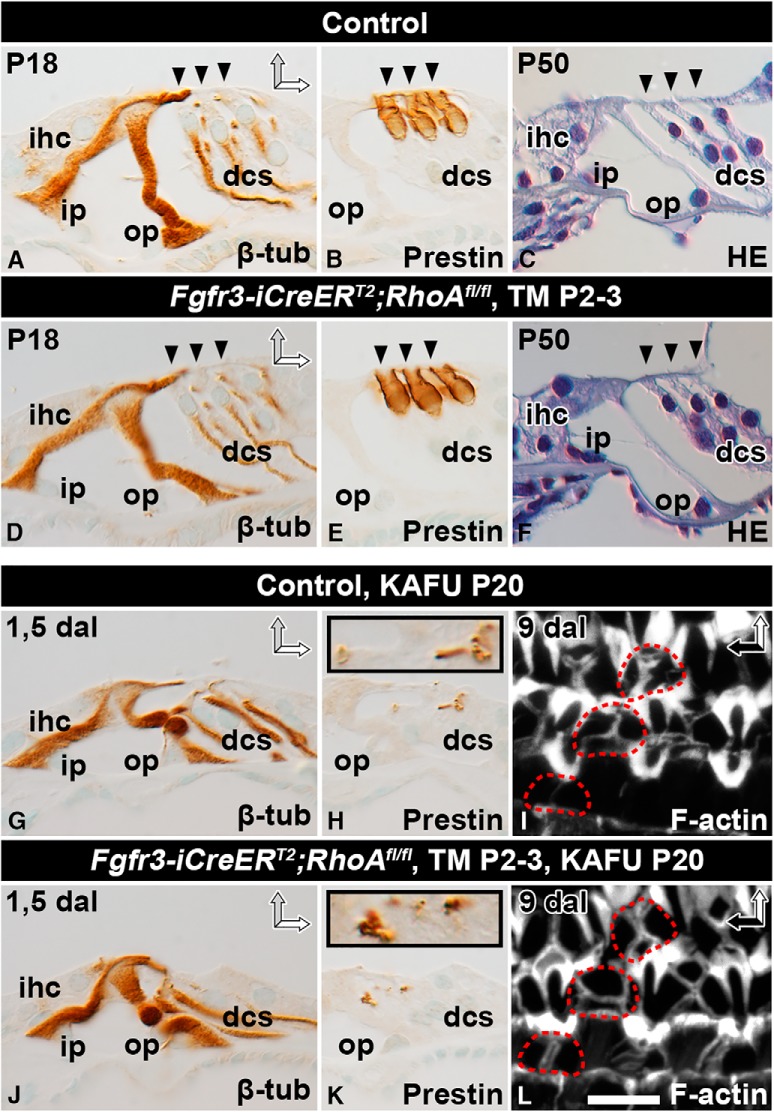
RhoA is dispensable for supporting cell maturation and wound healing. Recombination was induced in auditory SCs of *RhoA^fl/fl^;Fgfr3-iCre-ER^T2^* mice at P2–3, and analysis was performed at adulthood. Paraffin-embedded cross-sections (***A–H***, ***J***, ***K***) and phalloidin-labeled whole-mount specimens (***I***, ***L***) of the organ of Corti of the cochlear medial turn of mutant and control mice. ***A***, ***D***, At P18, β-tubulin immunocytochemistry shows no major structural differences between SCs of the two genotypes. ***B***, ***E***, Prestin immunostaining shows comparable morphology of non-recombined OHCs as well. ***C***, ***F***, Hematoxylin staining shows that the cytoarchitecture of the organ of Corti is comparable between mutant and control mice at P50. ***G***, ***J***, Most OHCs are lost 1.5 d after induction of ototoxic lesion (KAFU, see Methods). In both types of specimens, β-tubulin–positive SCs maintain positions at the reticular lamina, although their cell bodies have partially lost upright position. ***H***, ***K***, In both genotypes, Deiters cells phagocytose prestin-positive OHC debris, shown in insets at a higher magnification. ***I***, ***L***, Both control and *RhoA*-depleted SCs form stable F-actin scars, shown 9 d postlesion. Red dashed lines mark scars at the site of lost OHCs. Abbreviations: β-tub, β-tubulin; dal, days after lesion; dcs, Deiters cells; HE, hematoxylin; ihc; inner hair cell; i.p., inner pillar cell; KAFU, kanamycin and furosemide; *ohc*, outer hair cell; *op*, outer pillar cell; *TM*, tamoxifen. Scale (in ***L***): ***A–H***, ***J***, ***K***, 20 µm; ***I***, ***L***, 6.5 µm.

The apical stability of SCs could be experimentally modulated if the identity of molecules regulating their actomyosin cytoskeleton were known. A candidate molecule, RhoA, has been shown to regulate actomyosin dynamics and formation of cell–cell junctions in several cell contexts. Our *in situ* mRNA hybridization experiments demonstrated ubiquitous and low-level expression of *RhoA* mRNA in the cochlea (data not shown), as shown in other tissues (Pedersen and Brakebusch, 2012). To reveal RhoA’s function in postnatal Deiters and pillar cells, we induced *RhoA* inactivation selectively in these cell types from P2 onward in *Fgfr3-iCre-ER^T2^;RhoA^fl/fl^* mutant mice. Analysis of β-tubulin–immunostained sections and hematoxylin-stained sections at P18 and P50 revealed comparable SC morphology in mutant and control mice (Fig. 4*A*, *C*, *D*, *F*). Also, adjacent OHCs showed an unaltered phenotype, based on prestin immunostaining that marks the lateral plasma membrane of OHCs (Fig. 4*B*, *E*). This was expected, as postnatal OHCs remain unrecombined upon *Fgfr3-iCre-ER^T2^* activation (Anttonen et al., 2012). These results on sectioned material were confirmed in whole-mount specimens (data not shown).

RhoA is known to regulate actomyosin-dependent epithelial wound healing (Clark et al., 2009). Therefore, we next studied whether the morphology of *RhoA*-depleted Deiters and pillar cells is altered in ototoxically lesioned cochleas and whether these mutant cells can reseal the lesion and phagocytose the debris of dying OHCs as normally occurs (Anttonen et al., 2014). Recombination was induced by tamoxifen at P2 and P3 (Fig. 4*G–L*) or P16 and P17 (data not shown). Ototoxins were applied at P20, and analysis was performed 1.5 or 9 d postlesion. β-Tubulin immunostaining was used to visualize SC morphology and prestin immunostaining to mark the debris of dying OHCs. Both stainings were comparable between *RhoA*-depleted and control SCs (Fig. 4*G*, *H*, *J*, *K*). Phalloidin-labeled whole-mount specimens also showed that F-actin scars at lesion sites had a similar appearance in the two genotypes (Fig. 4*I*, *L*). Similarly, no differences were found between control and mutant mice when recombination was induced at P16 and P17 (data not shown). Thus, RhoA is dispensable for wound healing by adult SCs.

### RhoA is indispensable for outer hair cell development

Although we did not observe any effect of *RhoA* inactivation in postnatal SCs, RhoA might play a role in cell differentiation in the late-embryonic organ of Corti. To study this, we induced recombination in *Fgfr3-iCre-ER^T2^;RhoA^fl/fl^* mice starting at E13.5 and analyzed cochleas at E18.5. Between E13 and E14, prosensory cells of the presumptive organ of Corti exit the cell cycle and initiate differentiation as HCs or SCs. *Fgfr3* starts to be expressed in OHCs and SCs at this stage, and the resulting expression pattern is maintained until birth (Hayashi et al., 2010). Thus, in addition to Deiters and pillar cells, this late-embryonic recombination paradigm also targets OHCs (Kirjavainen et al., 2015).

**Figure 5.**
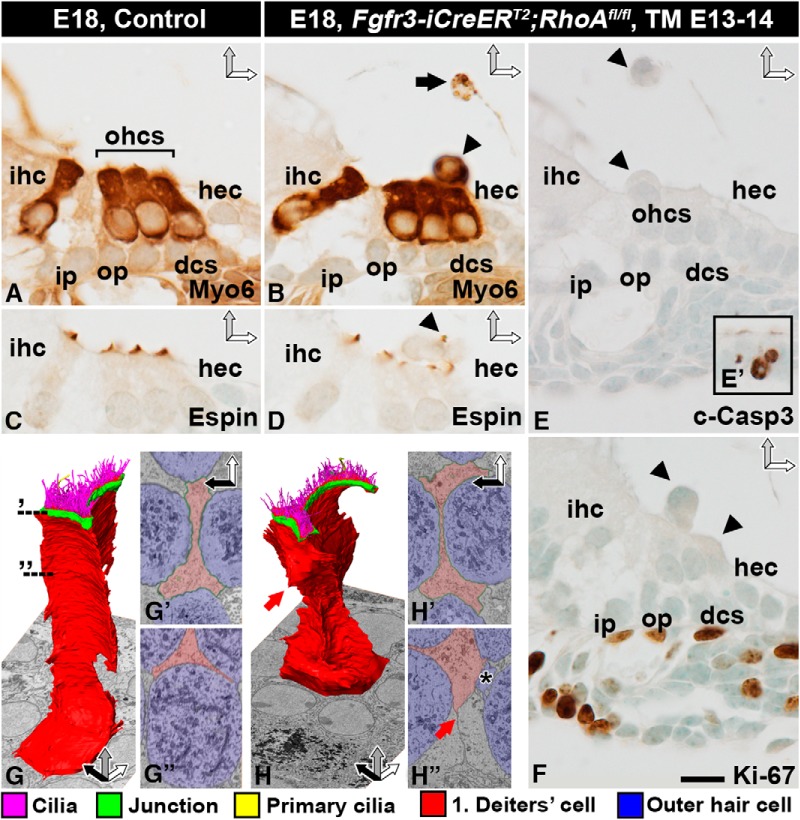
*RhoA-*depleted outer hair cells are extruded from the organ of Corti. Recombination was induced in *RhoA^fl/fl^;Fgfr3-iCre-ER^T2^* mice at E13–14, and analysis was performed at E18.5. Images show paraffin-embedded cross-sections from the medial turn of the cochlea (***A–F***) as well as single block-face images and 3D modeling of Deiters cells (***G–H″***). ***A***, ***B***, Myosin 6–immunostained OHCs extrude (arrowhead in ***B***) from the organ of Corti of mutant mice. The presence of myosin 6–positive degenerating cell profiles (arrow in ***B***) in the endolymph shows that OHCs die after extrusion. ***C***, ***D***, Similar to controls, OHCs of mutant cochleas possess espin-positive stereociliary bundles with abnormal morphology. Arrowhead points to an extruding OHC with an espin-stained bundle (***D***). ***E***, ***E′***, Extruding OHCs (arrowheads) are negative for cleaved caspase-3. As a positive control in the same section, the inset shows apoptotic cell profiles in the greater epithelial ridge of the cochlea. ***F***, Extruding OHCs are negative for Ki-67. Proliferating mesenchymal cells underneath the basement membrane in the same section serve as positive controls. ***G–G″***, 3D modeling of a control Deiters cell. Dashed lines demonstrate the level of block-face single images (***G′***, ***G″***). ***H–H″***, 3D modeling of a mutant Deiters cell shows that the general features of this cell type are maintained, but apical junctions (***H′***) and the basolateral cell membrane (red arrow in ***H***, ***H″***) have moved due to the loss of adjacent OHCs. A Deiters cell has invaded the space previously occupied by an OHC, and only a “tail” (asterisk) is left of the extruding OHC (***H″***). Color coding is explained below SBEM images. Abbreviations: c-Casp3, cleaved caspase-3; dcs, Deiters cells; hec, Hensen cell; ihc; inner hair cell; i.p., inner pillar cell; Myo6, myosin 6; ohc, outer hair cell; op, outer pillar cell; TM, tamoxifen. Scale bar (in ***F***): ***A–F***, 10 µm; ***G***, ***H***, 4 µm; ***G′***, ***G″***, ***H′***, ***H″***, 3 µm.

We prepared paraffin sections as well as SBEM and whole-mount specimens of *Fgfr3-iCre-ER^T2^;RhoA^fl/fl^* mutant and control cochleas at E18.5. Cross-sections through the mutant organ of Corti revealed an unaltered SC phenotype, whereas OHCs displayed distinct abnormalities (Fig. 5*A–F*). Several myosin 6–positive OHCs were undergoing extrusion from the epithelium (Fig. 5*A*, *B*), with some of them already extruded into the endolymph (Fig. 5*A–F*). The apex of mutant OHCs displayed espin-positive stereociliary bundles with an irregular appearance (Fig. 5*C*, *D*). The nuclei of extruding OHCs appeared intact. These cells were also negative for cleaved caspase-3, suggesting that apoptosis did not trigger their extrusion (Fig. 5*E*, *E′*). The mutant cells did not show cell cycle reentry, based on Ki-67 immunostaining (Fig. 5*F*). The presence of vacuolized and myosin 6–positive cell profiles in the scala media suggested that extruded OHCs degenerate in the endolymph (Fig. 5*B*). To confirm that the phenotype of adjacent Deiters cells was unaltered, we prepared 3D models from SBEM datasets. These models as well as block-face single images did not show any major alterations in Deiters cells. The morphology of these cells, however, was indirectly affected by the extrusion of adjacent OHCs (Fig. 5*G–H″*).

### RhoA targets perijunctional myosin II in developing outer hair cells

**Figure 6.**
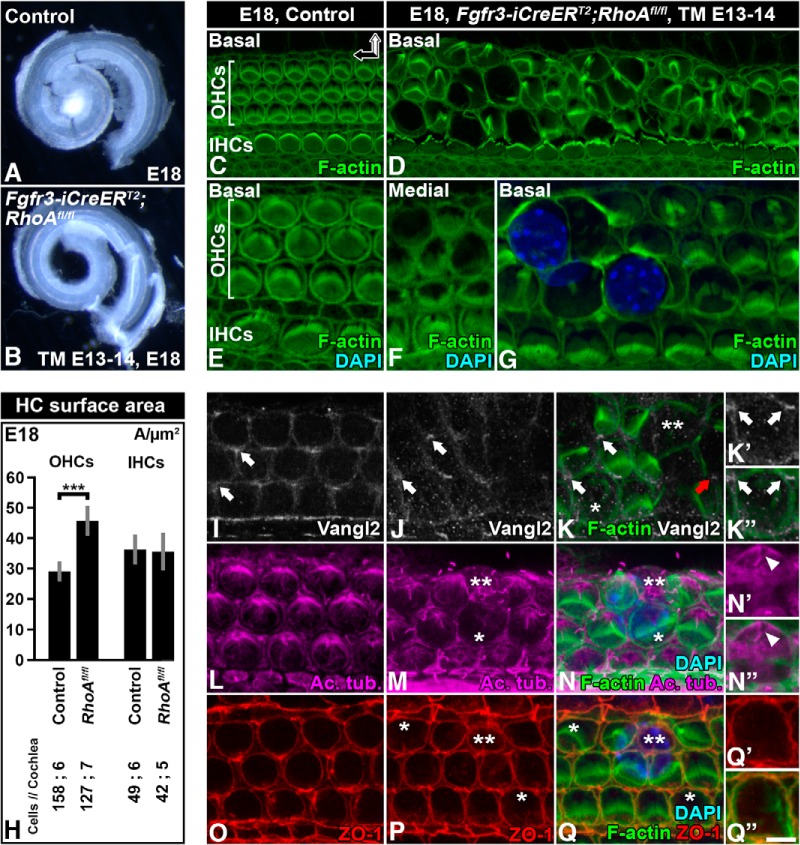
Loss of apical stability of *RhoA*-depleted outer hair cells and its effect on planar cell polarity, microtubule network, and junctional stability. Recombination was induced in *RhoA^fl/fl^;Fgfr3-iCre-ER^T2^* mice at E13–14, and analysis was performed at E18.5. ***A***, ***B***, Cochlear ducts of control and mutant mice viewed under dark-field illumination show comparable lengths. ***C***, ***D***, Compared with the control specimen, basal turn of the mutant cochlea shows distinct abnormalities in the apical domain of OHCs, revealed by phalloidin labeling. ***E–G***, Shown at a higher magnification and compared with the control specimen (***E***), medial turn of the mutant cochlea shows rather mild expansion of the OHC apical surface area. Hair bundles appear relatively normal as well (***F***). In contrast, the OHC apical area displays distinct expansion in the basal turn. Extrusion of OHC nuclei is evident (***G***). ***H***, Quantification of the width of the apical surface area of hair cells in the basal turn of control and mutant cochleas. Differences are statistically significant in the case of recombined OHCs, as opposed to non-recombined inner hair cells. ***I***, In controls, Vangl2 is expressed in the contact sites between the OHC’s medial wall and Deiters cells (arrows). ***J***, ***K***, In mutants, Vangl2 expression is maintained until OHCs are extruded (red arrow), based on phalloidin and Vangl2 double-labeling. ***K′***, ***K″***, An OHC with expanded apical surface is contacted by Vangl2-positive domains, shown at a high magnification. ***L***, Acetylated tubulin-positive microtubules radiate from the kinocilia in OHCs of the control specimen. ***M***, ***N***, In mutant OHCs with a slightly expanded apical surface area, microtubules appear to be largely intact. Microtubules become disorganized along with further expansion of the OHC apex (asterisk) and cell extrusion (two asterisks). ***N′***, ***N″***, In a mutant OHC, acetylated tubulin and phalloidin double-labeling shows an intact appearance of microtubules around the kinocilium (arrowhead). Microtubules become less organized along radiation toward the cortex. ***O–Q***, Similar to control OHCs (***O***), ZO-1 localizes evenly to tight junctions of mutant OHCs with an expanded apical surface area (asterisks; ***P***, ***Q***). This junctional expression is also seen in extruding OHCs (two asterisks; ***P***, ***Q***). ***Q′***, ***Q″***, A higher-magnification view of ZO-1–labeled tight junctions of an expanded OHC, shown together with phalloidin labeling. Images in ***I–Q″*** are from the medial turn of the cochlea. Abbreviations: Ac. tub, acetylated tubulin; TM, tamoxifen. Scale bar (in ***Q″***): ***A***, ***B***, 0.3 mm; ***C***, ***D***, 8 µm; ***E–G***, ***I–K***, ***L–N***, ***O–Q***, 5.5 µm; ***K′***, ***K″***, ***N′***, ***N″***, ***Q′***, ***Q″***, 3.5 µm.

We next examined the mechanisms behind OHC extrusion in *Fgfr3-iCre-ER^T2^;RhoA^fl/fl^* mice. At E18.5, the length of the cochlear duct was comparable between mutant and control animals, ruling out RhoA as a major effector of convergent extension-based cochlear growth (Fig. 6*A*, *B*; Wang et al., 2005). Phalloidin-labeled whole-mount specimens of control cochleas demonstrated the positioning of OHCs into three rows (Fig. 6*C*). This OHC patterning was clearly disturbed in mutant cochleas, most prominently in the basal turn (Fig. 6*D*). In the medial turn of *RhoA* mutant cochleas, most OHCs had an enlarged apical surface area. In the basal turn, the enlargement was more severe. In this segment, several OHC nuclei had risen above the level of the reticular lamina, and some OHCs were extruded as whole cells (Fig. 6*E–G*). Because cellular differentiation progresses in a basal-to-apical turn gradient along the length of the cochlear duct, and because the severity of the observed morphologic defects of *RhoA*-depleted OHCs worsened along this gradient, our findings link the altered OHC phenotype to their differentiation process.

**Figure 7.**
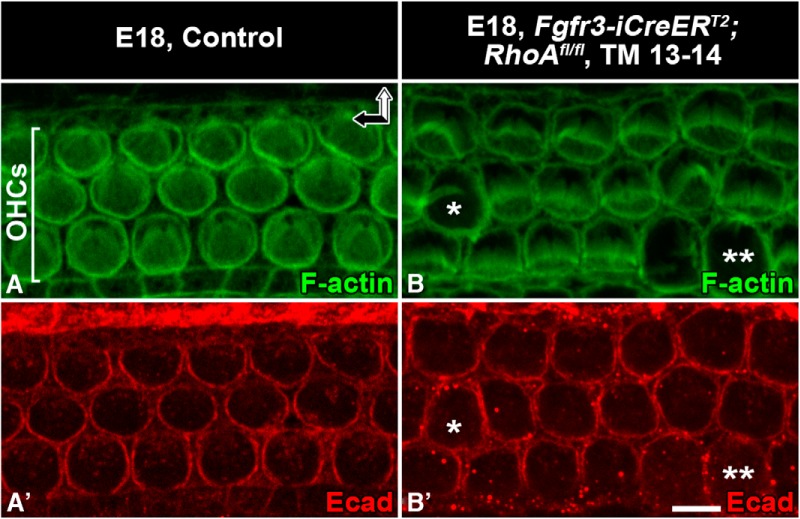
*RhoA*-depleted outer hair cells show normal junctional E-cadherin expression before their extrusion. Recombination was induced in *RhoA^fl/fl^;Fgfr3-iCre-ER^T2^* mice at E13–14, and analysis was performed at E18.5. Whole-mount specimens from the medial turn are shown. ***A***, ***A′***, The apical surface of control OHCs is delineated by E-cadherin–positive adherens junctions and a perijunctional actin ring, revealed by phalloidin labeling. Note the constant size of the apical cell surface areas. ***B***, ***B′***, In mutants, E-cadherin is still expressed in OHCs with expanded apical surface area (asterisk). In OHCs that are in the late phase of extrusion, E-cadherin is unevenly localized to junctions (two asterisks). Abbreviations: OHC, outer hair cell; Ecad, E-cadherin. Scale bar (in ***B′***): **A–B*′***, 5 µm.

We next studied whether planar cell polarity (PCP) is altered in OHCs of *Fgfr3-iCre-ER^T2^;RhoA^fl/fl^* mice. PCP is manifested in the cochlea as uniformly oriented HC stereociliary bundles. It is regulated by the core PCP proteins that localize to specific plasma membrane domains (Ezan and Montcouquiol, 2013). The kinocilium, located at the vertex of the V-shaped stereociliary bundle, and the associated microtubule network are instrumental for the establishment of PCP (Jones et al., 2008). In *Fgfr3-iCre-ER^T2^;RhoA^fl/fl^* mice at E18.5, stereociliary bundles of extruding OHCs had an abnormal appearance. However, bundle morphology was normal before OHC extrusion, at the stage when these mutant cells showed an enlarged apical surface area (Figs. 6*E–G* and 7*A*, *B*). In control specimens, the core PCP protein Vangl2 was expressed in the contact sites between the medial side of OHCs and Deiters cells (Fig. 6*I*), as has been described (Ezan et al., 2013). Comparable expression was seen in mutant specimens, despite altered geometry of the OHC apical domain (Fig. 6*J–K″*). Based on acetylated tubulin immunostaining, the kinocilium was correctly positioned at the vertex of the stereociliary bundle in mutant OHCs. Microtubule radiation from the base of the kinocilium was unaltered as well (Fig. 6*L–N″*). However, the distal ends of microtubules appeared to be partially delocalized from the perijunctional domain in OHCs with an enlarged apical surface area. This defect became more prominent along the progression of OHC extrusion (Fig. 6*M*, *N*). Taken together, this analysis did not reveal obvious PCP defects in the *Fgfr3-iCre-ER^T2^;RhoA^fl/fl^* mice.

To reveal possible effects of *RhoA* inactivation on junctional proteins, we investigated the expression of ZO-1 (Fig. 6*O–Q″*) and E-cadherin (Fig. 7*A′*, *B′*) in OHCs of *Fgfr3-iCre-ER^T2^;RhoA^fl/fl^* mice at E18.5. As in controls, the apex of mutant OHCs was evenly bordered by the expression of both junctional components, although their surface area had expanded. These expressions became nonuniform only after OHC extrusion was almost complete (data not shown).

**Figure 8.**
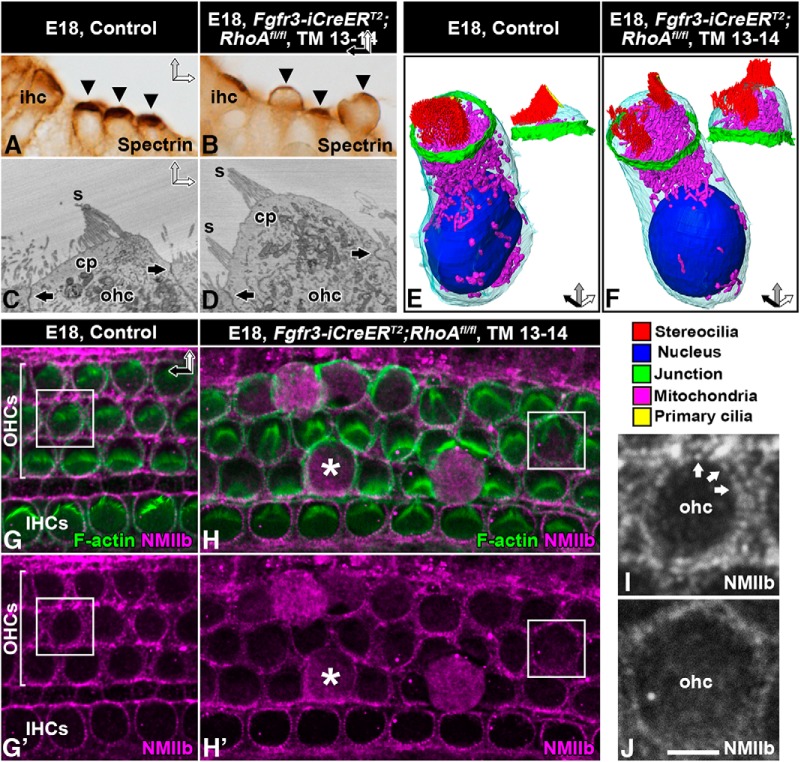
Loss of apical stability of *RhoA*-depleted outer hair cells and altered intracellular NMII localization. Recombination was induced in *RhoA^fl/fl^;Fgfr3-iCre-ER^T2^* mice at E13–14, and analysis was performed at E18.5. Images show paraffin-embedded cross-sections (***A***, ***B***), block-face single images (***C***, ***D***), 3D models (***E***, ***F***), and whole-mount specimens (***G–J***) from the medial-basal region of the cochlea. ***A***, ***B***, Spectrin immunostaining marks cuticular plates (arrowheads). Cuticular plates of mutant OHCs bulge outward, as opposed to control ones. ***C***, ***D***, Control OHC shows apical junctions (arrows) and a linear cuticular plate that anchors stereocilia (***C***). Mutant OHC shows intact junctions (arrows). Its cuticular plate bends upwards, splitting the stereociliary bundle (***D***). ***E***, ***F***, 3D modeling confirms these phenotypic changes. The morphology of mitochondria appears comparable in control and mutant OHCs. Color coding is shown below images. ***G***, ***G′***, Double-labeling for F-actin and NMIIb shows perijunctional colocalization in control OHCs. Note the puncta-like expression of NMIIb. Squares in ***G***, ***G′*** mark the cell shown in ***I***. ***H***, ***H′***, Along with expansion of the apical surface of mutant OHCs, NMIIb delocalizes from the cortex into diffuse cytoplasmic expression (asterisk). Note that cortical NMIIb localization is maintained in non-recombined inner hair cells. Squares in ***H***, ***H′*** mark the cell shown in ***J***. ***I***, ***J***, Higher magnification views of boxed areas in ***G***, ***G′*** and ***H***, ***H′***, respectively. Cortical NMIIb is expressed in a puncta-like pattern (arrows) in the control OHC (***I***). Mutant OHC lacks the strictly cortical expression of NMII and its puncta-like pattern (***J***). Note that this image shows a *RhoA*-depleted OHC before its extrusion from the epithelium. Abbreviations: cp, cuticular plate; ihc, inner hair cell; ohc, outer hair cell; s, stereocilia; TM, tamoxifen. Scale bar (in ***J***): ***A***, ***B***, 10 µm; ***C***, ***D***, 3 µm; ***E***, ***F***, 5 µm; ***G–H′***, 8.5 µm; ***I***, ***J***, 3 µm.

HCs contain a unique subapical actin-spectrin matrix, the cuticular plate, which serves as an anchoring site for stereocilia. In cochlear sections from *Fgfr3-iCre-ER^T2^;RhoA^fl/fl^* mice at E18.5, OHCs displayed spectrin-immunolabeled cuticular plates that were abnormally thin and were bent upwards (Fig. 8*A*, *B*). Single block-face images and 3D modeling from SBEM datasets confirmed these findings (Fig. 8*C–F*). In addition, these datasets revealed that the stereociliary bundles of extruding OHCs were split into small islands, consistent with phalloidin labeling in whole-mount specimens (Fig. 6*D*, *G*).

RhoA regulates the dynamics of the perijunctional actomyosin network at the apices of epithelial cells (Quiros and Nusrat, 2014). Phalloidin labeling demonstrated the maintenance of F-actin in this perijunctional area of *RhoA*-depleted OHCs (Figs. 6 and 7*A*, *B*). Prior studies have localized NMII isoforms b and c to the perijunctional area of late-embryonic and neonatal OHCs (Yamamoto et al., 2009; Ebrahim et al., 2013). We confirmed this expression of NMIIb in control OHCs at E18.5. In contrast, mutant OHCs showed NMIIb delocalization to the cytoplasm (Fig. 8*G–J*). This diffuse cytoplasmic expression became more pronounced as the apical surface expanded and as cell extrusion progressed (Fig. 8*H*). Thus, RhoA is essential for the maintenance of perijunctional localization of NMII and appears to promote tensional homeostasis of differentiating OHCs.

## Discussion

The perijunctional actin cytoskeleton regulates epithelial cell shapes and their positioning. It is of considerable interest to understand how cells with an overly rigid actin cytoskeleton are able to move when necessary. During wound healing, shape changes and movement of these kinds of cells are limited, unlike cells with a nonrigid actin cytoskeleton. Another related question is the role of the perijunctional actomyosin network in providing rigidity to cells to oppose external and internal forces. Both questions are crucial for understanding the development and adult functions of SCs and HCs, cell types with specialized apical actin cytoskeletons. Here, we provide direct evidence for unique wound healing mechanisms by the thick F-actin belts of mature auditory SCs. We also show that RhoA participates in the molecular regulation of perijunctional stability in developing OHCs. These findings have translative importance regarding the restoration of hearing function. For interventions that seek to regenerate HCs, it is important to consider that proper development of the apical actomyosin cytoskeleton is required for HC functionality. Furthermore, regenerative and protective approaches should take into account that cellular patterning and barrier integrity of the adult organ of Corti should be maintained for it to remain functional. The apical actin cytoskeleton is critically involved in both of these events.

### Thick F-actin belts of supporting cells maintain dimensions of the apical surface of the lesioned organ of Corti

Mechanisms of wound repair have mostly been studied *in vitro* in more simple epithelia that comprise cells with thin F-actin belts. Lesions in these kinds of epithelia are generally closed by a multicellular contractile actomyosin purse string, involving shape changes and movement of cells bordering the lesion site (Clark et al., 2009; Sonnemann and Bement, 2011). This has been demonstrated in explant cultures of the developing utricle, where wound healing depends on the shape changes of SCs, made possible by their thin F-actin belts that contribute to the formation of a multicellular purse string (Burns and Corwin, 2014). In contrast, no purse string was formed by the thick-belted SCs of the adult utricle, despite successful wound closure (Burns and Corwin, 2014). The present study focuses on the thick F-actin belts of auditory SCs. We show that the thick-belted SCs of the adult organ of Corti stay motionless during wound healing, and therefore, the width (medial-lateral) and cellular patterning at the reticular lamina remain unaltered. In contrast, the width and cellular patterning at the surface of the developing organ of Corti are dramatically altered on wound healing, because thin-belted SCs cannot resist shape changes. Thus, the thick F-actin belts serve as multicellular scaffolds that hold the apical cell domains in place during wound healing. This cytoskeletal adaptation appears to be of utmost importance for the hearing function that is dependent on the correct cytoarchitecture of the organ of Corti. Interestingly, the junctional domain between Deiters cells and OHCs forms a unique hybrid structure where the tight and adherens junction proteins colocalize to a single large junction (Nunes et al., 2006). Thus, the whole junctional/F-actin entity appears to provide unique stability to the reticular lamina, which enables the organ of Corti to withstand sound-induced mechanical forces and maintain cellular topology during wound healing.

### Unique wound healing mechanisms in the adult organ of Corti

We provide direct evidence that the strong F-actin belts of mature SCs force unique mechanisms of wound healing. On OHC loss, when SC apices expand to the lesion site, junctional components failed to detach from the original F-actin belts: large junctional protein plaques remained in association with the original belts and did not contribute to the formation of new SC–SC junctions. Our results are supported by prior data showing the concurrent presence of the previous SC–OHC junctions in their original position together with new SC–SC junctions at the lesion site (Raphael and Altschuler, 1991; Leonova and Raphael, 1997). Our data suggest that reduced plasticity of the junctional domain of SCs is compensated for by protruding activity of the basolateral domain of the same cells. We found that even more distant SCs participated in the formation of new junctions by the formation of basolateral membrane protrusions. The noncompact lumenal structure of the organ of Corti appears to allow the growth of long protrusions to the lesion site. This might not be possible in epithelia lacking lumenal spaces, such as the vestibular sensory epithelia. However, the participation of distant SCs in wound healing in the organ of Corti seemed to be restricted to the most acute wound healing phases, as we could not document it in later phases. Nevertheless, its existence suggests that wound healing is triggered by diffusible signals, i.e. no previous physical contact between a SC and a dying HC is needed to initiate repair. ATP and Ca^2+^ released from dying HCs have been suggested to play a role in wound healing in the organ of Corti (Gale et al., 2004; Lahne and Gale, 2010), but the identity of diffusible signals involved in this process remains to be conclusively characterized.

### RhoA is dispensable for wound healing and maturation of supporting cells

In search for regulators of wound healing in the organ of Corti, we have used genetic approaches to inactivate *Rho* GTPases. It has previously been shown that Cdc42 is essential for the maturation of the apical actin cytoskeleton of early postnatal auditory SCs. These maturational defects caused impaired wound healing at adulthood. However, when *Cdc42* inactivation was induced in SCs at the adult stage, wound healing was unaffected (Anttonen et al., 2012). The current results show that both the maturation and wound healing capacity of SCs are unaffected by *RhoA* inactivation. Considering the established role of RhoA in wound repair in various cellular contexts, our results seemed at first glance surprising. On the other hand, prior data show that wound healing by adult utricular SCs does not depend on the formation of the actomyosin purse string (Burns and Corwin, 2014). In that light, our results appear less unexpected.

### RhoA regulates actomyosin/junctional stability of developing outer hair cells

Previous studies demonstrated that RhoA promotes actomyosin contractile activity of cultured cells (Benink and Bement, 2005; Jackson et al., 2011; Sonnemann and Bement, 2011). *In vivo* studies with *RhoA*-depleted progenitor cells also point to this role. For example, *RhoA*-inactivated epithelial progenitor cells failed to apically constrict during invagination of the lens and otic placodes, as evidenced by impaired actomyosin contractility and increased apical cell surface area (Chauhan et al., 2011; Sai et al., 2014). By focusing on later stages of development of epithelial cells, we show here that auditory SCs are not affected by *RhoA* inactivation. In contrast to SCs, depletion of *RhoA* in adjacent developing OHCs led to striking abnormalities in their apical domain. This shows that the requirement of RhoA for apical domain stability differs radically between cell types, even between cell types of the same epithelium.

The enlargement of the apical surface areas of developing *RhoA*-depleted OHCs, followed by the extrusion of these cells from the epithelium, suggests that OHCs require RhoA for cortical tension maintenance. The apical domain of mutant OHCs appeared to be unable to oppose internal forces, in which the growing cuticular plate may play a crucial role. The cuticular plate itself seemed not to be a direct target for *RhoA* inactivation: the apical surface area of OHCs was expanded before the formation of a visible cuticular plate. This suggests that the altered phenotype had its origin in the cell cortex.

A previous study showed that *RhoA* depletion in lens progenitor cells impaired apical constriction, which was associated with NMII downregulation (Chauhan et al., 2011). In contrast, our results suggest that RhoA signaling promotes the maintenance of perijunctional stability of developing OHCs by regulating the localization of NMII: NMII expression was maintained but delocalized from the perijunctional domain into the cytoplasm in *RhoA*-inactivated OHCs. This indicates disturbed coupling between the actomyosin network and junctional proteins (Etournay et al., 2010; Ebrahim et al., 2013; Quiros and Nusrat, 2014). In neuroepithelial progenitors, *RhoA* inactivation caused disruption of junctional proteins (Herzog et al., 2011; Katayama et al., 2011). This was not seen in *RhoA*-depleted OHCs, except at late phases when OHCs extruded out from the epithelium. Thus, it appears that RhoA is not critical for the maintenance of cell–cell junctions in the organ of Corti *in vivo*. A previous study has also demonstrated that *RhoA* inactivation in mature cells does not disrupt cell–cell junctions *in vivo*: mature keratinocytes depleted of *RhoA* lacked junctional disturbances. However, when these mutant cells were isolated into culture conditions, *de novo* formation of cell–cell junctions was impaired (Jackson et al., 2011).

Consistent with the suggestion that *RhoA* inactivation induces apical surface enlargement in OHCs by limiting the activity of junctional NMII, previous *in vitro* studies showed that treatment of developing cochlear explants with the NMII inhibitor blebbistatin increases the HC apical surface area and impairs tensional homeostasis (Yamamoto et al., 2009; Ebrahim et al., 2013). Contrary to our results, these studies did not document OHC extrusion, perhaps because of different experimental setups (*in vitro* versus *in vivo*), different age [NMII inhibition initiated at P1 in Ebrahim et al. (2013)], or different strength or specificity of inhibition by pharmacological versus genetic means.

The final fate of developing *RhoA*-inactivated OHCs appeared to be whole cell extrusion from the organ of Corti into the endolymph. Extruding OHCs did not show caspase-3 activation nor expression of Ki-67, demonstrating that the altered phenotype was not linked to apoptotic degeneration or increased proliferative capacity. These results suggest that OHC extrusion, induced by *RhoA* inactivation, is a cell-intrinsic process. However, although we did not find morphologic changes in neighboring SCs, we cannot rule out the possibility that subtle changes in these cells contribute to the altered OHC phenotype. Altogether, our results suggest that RhoA is essential in differentiating OHCs for the cohesion of the perijunctional actomyosin network and its coupling with the junctional domain.

As *Cdc42* (Kirjavainen et al., 2015), *Rac1* (Grimsley-Myers et al., 2009), and *RhoA* (this study) have now been inactivated in the developing organ of Corti, we can compare the roles of these prototypical members of the Rho GTPase family. Inactivation of both *Rac1* and *Cdc42* triggered patterning defects in the late-embryonic organ of Corti, including PCP defects, i.e., misorientation and dysmorphology of the HC stereociliary bundles (Grimsley-Myers et al., 2009; Kirjavainen et al., 2015). In addition, *Rac1* inactivation caused defects in the global growth of cochlea, suggesting disturbances in convergent extension (Grimsley-Myers et al., 2009). We did not observe these defects after *RhoA* inactivation, although RhoA’s major effector NMII has been shown to regulate convergent extension during cochlear development (Yamamoto et al., 2009). Compensatory effects of other Rho GTPases or the spatial/temporal gene inactivation strategy used might explain why we did not see defects in convergent extension of the cochlear duct of *RhoA* mutant mice.

To conclude, we have found, first, that the thick F-actin belts of SCs are essential for the maintenance of dimensions and positioning of cell apices in the lesioned organ of Corti. Second, SCs can be recruited to wound healing independently of their physical contact with dying OHCs. These results underline the importance of maintaining an intact SC population in future regenerative interventions. Third, we show that RhoA is dispensable for wound repair by auditory SCs, but it plays a role in the regulation of actomyosin stability at the apical domain of developing OHCs.

## Synthesis

Reviewing Editor: Darcy Kelley, Columbia University Decisions are customarily a result of the Reviewing Editor and the peer reviewers coming together and discussing their recommendations until a consensus is reached. When revisions are invited, a fact-based synthesis statement explaining their decision and outlining what is needed to prepare a revision will be listed below. The following reviewer(s) agreed to reveal their identity: Bernd Fritzsch

Both reviewers found this paper to be a valuable contribution to the literature and I concur. I have synthesized their findings and recommendations as follows:

This works improves understanding of how supporting cells in the adult mouse cochlea mobilize to seal vacancies left by dead hair cells. Basic observations such as these serve as the foundation for more detailed, molecular level hypotheses. The authors test one such molecular level hypothesis around the role of RhoA in actin dynamics and wound healing. This is a first detailed description of the effect of RhoA in the early phase of organ of Corti degeneration. The findings were negative, but it is important for the field to know that RhoA is dispensable in this process. In addition, the tools the authors created to test their hypothesis did lead to the discovery of an important role for RhoA in hair cell development. Thus, the field should find at least three different aspects of this manuscript useful.

While the paper is overall well constructed and illustrated, the writing lacks clarity in parts due to unusual sentence constructions and word choices. The paper's impact might be enhanced by outlining the final outcome of hair cell elimination in the introduction to provide a clearer framework for the analysis

This manuscript describes very elegant work examining the complex movements of cochlear supporting cells as they repair the damaged sensory epithelium, the consequences of these movements on their apical junction complexes, and the role (or lack thereof) of RhoA GTPase which is well-known to contribute to wound healing in other systems. While they find that RhoA is not essential for supporting cells to seal lesions, they do uncover a requirement for it during hair cell development. The IHC and SBEM analysis is superb, and the authors do an excellent job of presenting some of the more complicated block-face images in an easy to interpret manner.

A few additional small experiments are needed:

1. The data showing remnants of junctional proteins associated with preexisting F-actin belts is intriguing. To bolster the depth of this conclusion, two additional pieces of evidence should be collected: 1) an image of ZO-1 labeling in a control cochlea, and 2) labeling of other junctional proteins in a damaged cochlea. ZO-1 is an adaptor molecule, not a membrane protein like other components of the junctional complex. It seems less surprising to find residual adaptor proteins associated with the original F-actin belts, but what about membrane proteins like E-cadherin or Occludin?

2. While the expression pattern of CreER has previously been characterized in the Fgfr3- iCreER mouse, the targeted, conditional deletion of RhoA needs to be confirmed at the protein level at minimum.

Detailed Comments

1. Introduction, line 5. Deiter's and pillar are only some of the supporting cells. The authors may want to be precise here. The authors might check a recent review (Jahan et al., 2015) for details on supporting cells.

2. Intro, last paragraph. The authors should add to Rho function the other GTPases known to play a role in actin homeostasis (Rac, Cdc42). Cdc42 is perhaps the only other GTPase with a known function in the organ of Corti:loss of hair cells with a bias toward IHCs (Ueyama et all , J Cell Biol, 2014; Kijavainene et al., Biol Open, 2015; Grimsley-Meyers et al., 2000)).

3. Material and Methods

a. First paragraph. The convention is to consider noon of the day of the plug to be E0.5 (Theiler, 1987)

b. Tmx injection. Why was injection chosen over gavage? Previous work has demonstrated that gavage is more reliable as IP injections can leak out some of the injected fluid.

c. Specimen prep for SBEM> A citation is needed after .....described in detail (.....).

4. Approach

HC death via Gfi1 LOF is not the same as HC death via KAFU. Gfi1 LOF targets HCs specifically, whereas SCs and other cells within the cochlea are exposed to KAFU, which could be detrimental to their health in ways that are not grossly visible in sections. In addition, Matern et al. (Sci. Rep., 2017) recently showed that Gfi1 is expressed by resident macrophages in the neonatal cochlea. Thus, it is possible that Gfi1 LOF impairs macrophages that could play a yet to be defined role in this process. If the KAFU treatment is not possible in neonates, the authors should describe limitations of the comparison between the two methods in more detail.

5. Results

a.The findings presented in the first four sections of the Results are compared to similar findings in the adult mouse utricle. One potential important difference between the supporting cells in these two organs is the “tight-adherens” junctions in the adult cochlea, which have been described by Nunes et al., J. Cell Sci., 2006. It would be good to discuss the findings in the context of this unique structure.

b. Do the authors know if deletion of RhoA at earlier timepoints has a more subtle effect on supporting cells that could be contributing to the HC phenotype? If not, this should be discussed.

c.Second paragraph. A more detailed description of cellular processes of translating the PCP signaling into cell type specific action and how to reorganize microvilli into stereocilia as revealed in more recent work is needed (May-Simera et al., Development 2015; Tarchini et al., Development., 2016).

d.Since no mechanical measurements are made in this study, the authors should avoid any conclusions about mechanical properties of the cells (e.g. p. 9: “Thus, the thick F-actin belts of mature SCs provide rigidity for the maintenance of positioning of cell apices.”). “Stability” would be more appropriate terminology.

e. p.10, “While increased rigidity of the apices of adult SCs maintains dimensions of the organ of Corti, it should not limit the wound healing process itself.” Why wouldn't rigidity (or stability) limit the wound healing process?

6.Discussion

a.First paragraph. The end product of degeneration is the flat epithelium that seems to come about through replacement of the organ of Corti by cells overgrowing it from the lateral wall no matter the way hair cells are eliminated (Taylor et al., 2012; Kersigo et al., 2015) and in embryos these 'replacement cells' appear to be the BMP4 posiitve Claudius cells (Pan et al., 2012). It appears to be important to relate the final outcome of HC degeneration to readers up front.

b.Second to last paragraph. This discussion needs update with May-Simera et al and Tarchini et al insights. Both data sets do not relate the intracellular signaling leading to PCP translation into action to the RhoA signal.

Figures

Fig 1 Note that OHC1 are in contact with OP, IP, and D1; OHC2 with OP, D1, D2; OHC3 with D1,D2,D3. Since OP are different in their tubulin skeleton and IP/OP are the last cells to de-differentiate after HC loss, this should be pointed out. It is not clear why the replacing cells in G are refered to as Hensen's cells when ISH data suggest that these are likely Claudius cells (see Pan et al., 2012 on Atoh1 null mice).
